# Research on the Application of Polymer Materials in Contemporary Ceramic Art Creation

**DOI:** 10.3390/polym14030552

**Published:** 2022-01-29

**Authors:** Xiaobing Hu, Yuanqian Lai, Yingshuang Hu, Yingzhuo Li, Dan Zhao, Fang Tong

**Affiliations:** 1 Fine Art School, Anqing Normal University, Anqing 246001, China; 1976hxb@163.com (X.H.); e2388298909@hotmail.com (Y.H.); onew121478@outlook.com (Y.L.); z13235918997@hotmail.com (D.Z.); T768905708@hotmail.com (F.T.); 2 School of Art, Southeast University, Nanjing 210018, China

**Keywords:** polymer materials, modern pottery creation, vision and performance, three-dimensional printing

## Abstract

Ceramicized polymer composites are prepared by adding various additives into the polymer matrix, such as functional clay fillers, porcelain-forming additives, crosslinking agents, flame retardants, reinforcement agents, etc. In recent years, polymer materials have been widely used in the preparation of ceramic materials. Moreover, the addition of polymer materials in ceramic materials results in increased bending in the ceramic body, and its mechanical strength has been greatly improved; this advantage has led many contemporary ceramists to use polymer materials in the creation of ceramic works, providing ceramic creation more space for operation. The introduction of polymer materials into ceramic materials brings more possibilities than traditional ceramic creation based on the tests of toughness, strength, and yield of the ceramic body. This article investigates ceramic raw materials with high-polymer material ceramic function, high-polymer materials for modern pottery to convey the artistic expression of porcelain texture, as well as the use of high-polymer materials in gel-powered three-dimensional (3D) printing to refine the injection molding process, all of which solve the difficulties of creating delicate artworks in modern ceramic art creation. This paper mainly adopts the research method of recording and comparing the numerical value of adding ceramic materials into polymer materials and the physical shape after firing in ceramic creation, to form a relatively stable numerical value and firing curve for a certain type of ceramic creation form. In this regard, the integration of modern ceramic creation and polymer materials makes ceramic works a relatively special style in contemporary art, increasing its cultural connotation and visual tension.

## 1. Introduction

Polymer materials are one of the rapidly developed and more used protective materials in recent years, mainly including acrylate, epoxy resin, silicone, organic fluorine cellulose derivatives, etc. [1]. Professor Yibing C, Monash University, Australia, invented ceramic polymer composites, used for refractory cables and were commercially produced by Ce-ram Polymerik, Australia [2]. After research, Yuhong C, and other scholars considered the feasibility of cellulose–chitosan copolymer products for the sealing and protection of stone cultural relics and prepared cellulose–chitosan grafting copolymers [3]. After protection by the sealing material with cellulose–chitosan grafting polymer concentration, the stone sample maintains the original texture, luster, and color, and hydrophilicity, aging resistance, and other properties have been greatly improved, which has a broad prospect in ceramic art.

Due to the participation of polymers in ceramic production, in the process of ceramic production, the addition of ceramic enhancers can improve the strength of the initial porcelain products and also improve their lubricity, as a lubricant in the process of ceramic production can promote the formation of blank, prevent sticky mold, make the product easy to off mold, and improve the quality and yield of finished products. In the current ceramic production, the lack of edge drop phenomenon caused by the lack of blank strength is more serious, which reduces the yield rate. The preparation of blank is a very important link. Therefore, in the creation of modern ceramic art, how to improve the success rate of raw material tire formation and the visual appearance of ceramic works has become an urgent problem for contemporary ceramists and ceramic artists to solve. In recent years, many scholars have discussed the influence of polymer materials interventional ceramic raw materials on ceramic products in depth and mainly from several aspects. First, polymer materials in ceramic preparation of raw materials are mainly put forward since, with the improvement in people’s quality of life, traditional single stone, wood, glass, ceramics, paint, and other materials cannot meet people’s needs, and therefore, a large number of new decorative materials emerged and developed rapidly; this is reflected in varieties and types, but also reflected in the material nature, function, and grade, so features such as green environmental protection, superior function, luxury decoration, and other remarkable performance aspects of multifunctional composite decorative materials will be more favored by people [4]. Professor Wang and others believe that silica-based materials are one of the most widely used materials in optics, memS, aerospace, and some other industries [5]. In practical applications, silica-based materials need to be connected with themselves or other heterogeneous materials, to assemble products with various functions, and these researchers elaborated the future development trend of SiO_2_ ceramic connection, used to comprehensively understand the potential improvement of SiO_2_ ceramic connection technology. Areias et al. and other scholars believe that municipal sewage sludge can be directly added to clay bricks for construction. With Winkler and the prediction chart, it is revealed for the first time that the maximum addition is 50 wt%. Clay bricks produced at firing temperatures of 850 °C and 950 °C and up to 15% sludge addition have no environmental impact and meet the criteria of linear shrinkage, water absorption, and mechanical strength. In addition, sludge as fuel contributes 40% of the combustion energy in the sintering process. As a result, clay bricks with sewage added may be made at the lowest price, costing only 16 percent of the corresponding concrete bricks and 20 percent of ordinary clay bricks fired at higher temperatures [6]. Scholars such as Vilarinho believe that waste valuation is crucial because natural raw materials are scarce. In Europe, eggshell waste is produced up to 150 kroner/year, and landfills are often the only option. This research aimed to develop eco-ceramic wall tiles using biological calcium carbonate from eggshell waste as raw materials. Several pastes were prepared using eggshell wastes instead of the natural raw limestone used today. Total substitution of bio-calcium carbonate using the same particle size method resulted in a 19% increase in bending strength through the characterization of samples, without affecting other properties (linear thermal coefficient, weight reduction, emission shrinkage, water absorption, density, color coordinates), all values within the industrial range [7]. The application prospect of polymer materials in building ceramics is very broad, which will make an immeasurable contribution to improving people’s living environment and improving the quality of life [8,9,10]. In terms of firing and high-temperature resistance of ceramic products, some scholars propose that continuous fiber-reinforced ceramic-based composites have low density, high strength, high toughness, and good oxidation resistance and, therefore, have become competitive candidates.

Moreover, it is proposed that in ceramic products, different new antioxidant interface phases are needed to choose according to different fibers and ceramic matrix, and the preparation method is optimized to prepare continuous uniform and dense interface phases without damaging the fiber strength while improving the overall performance of the ceramic matrix composites [11]. However, due to the relatively weak interface strength of most CFCC and that the fracture process is more complex than traditional composites, exploring the fracture process and the factors affecting the fracture process is key to in-depth study and application of CFCC. Starting from the CFCC matrix defect, Hailong X reasonably simplified the defect to the lip defect, solved the theory solution of the defect stress strength factor (SIF) and the conformal transformation theorem, used it as the criterion for crack expansion, and further analyzed the impact of the geometric size of the defect on SIF [12]. Although this material may cause defects in the preparation of raw materials, it provides the vision of technical support for the creation of contemporary ceramic works. Second, the application of polymer materials is conducive to the 3D printing of ceramic products. The 3D printing method is used to model ceramic products in advance, and then, the design and modeling works are accurately produced through 3D printing equipment. As 3D printing can accurately realize the creative thinking of artists and designers, 3D printing technology is increasingly used in the creation of ceramic products and contemporary ceramic works. Moreover, given the difficulty of forming complex structural porous ceramics, long mold making cycle and high cost, a research group studied the forming process of porous ceramic materials based on 3D printing mold and gel injection mold forming. Based on making high-precision regular resin mold using light-curing molding technology, the low viscosity and high solid content alumina ceramic slurry required for the gel injection molding process is optimized, and the aluminum oxide ceramic billet is formed by the vacuum compression process, to achieve the net formation of porous ceramic parts of complex structure [13]. Pan and other scholars proposed that it is an attractive strategy to combine 3D printing technology with polymer ceramic technology to construct wave-absorbing honeycomb with fine structure through a flexible process. However, the preparation of honeycomb ceramics with superior mechanical and absorbing properties is still a challenge. Using polysilazane and multifunctional acrylate as raw materials, SiCN honeycomb ceramics were prepared by stereolithography. By optimizing the multifunctional acrylate and its ratio, the decomposition of organic components was matched with the ceramization process of the precursor, so that the ceramics had higher compactness [14]. It can be seen that domestic and foreign researchers have achieved much success in the preparation of polymer materials by adding ceramic raw materials and using 3D printing technology to form the mechanism of ceramic formation, the structural characterization of ceramic bodies, and the overall performance of ceramicized polymers. Although most of these research results focus on the production of materials and special ceramics, they provide many useful references for contemporary ceramic creation in terms of materials and molding. This article discusses the application of polymer materials in the creation of modern ceramic art. The purpose is firstly the fusion of polymer materials in ceramic raw materials to improve the strength of the ceramic body; secondly, it is helpful to increase the bending and toughness of ceramic creation, making it possible to create difficult modern ceramic art; thirdly, the use of polymer gel injection molding technology, combined with 3D printing molds, realizes the formation of complex shapes such as porous and multi-layered shapes. Porcelain craftsmanship is used to study the multiple functions and visual space of modern pottery creation. Through these three parts, the research aimed to use interdisciplinary thinking to explore the integration of modern ceramic art creation and the frontier fields of science and technology, and to play a guiding role in analysis, style, and paradigm for modern ceramic art creation.

## 2. Methods

This study mainly began from the following research methods:

The first was the on-site recording method. The fusion of polymer composite materials and ceramic raw materials involves the ceramic effect formed by different numerical ratios. The different ratio values are mainly recorded through the recording method. The ceramist marks the different ratio values. After the porcelain blank is naturally dried, The physical, stress, and finished product styles of the porcelain blanks are recorded in detail for use as a reference model.

The second was the sample burning method. Ceramics are made in three parts and fired in seven parts. The fusion of polymer composite materials and pottery raw materials. After the ceramic body is naturally dried, it must undergo trial firing to analyze the effect of different temperature curves, kiln positions, and time on the formation of ceramic bodies. The impact is ready to form a paradigm and improve yield.

The third method used was the cross-fusion method. Ceramic craftsmanship is a multi-disciplinary fusion. This study focused on combining the cross-fusion method with computer images, using computers to present creative ideas, and using 3D technology to solve difficult craftsmanship. The creative concept and design thinking were put into practice.

## 3. The Fusion of Polymer Materials and Ceramic Raw Materials Enhances the Artistic Function and Spatial Vision of Ceramics

Ceramics is a general term of pottery and porcelain, but also a form of arts and crafts in China. In the Neolithic Age, China had rough and simple, colored and black pottery. Tao and porcelain have different textures and different properties. Tao is made of high viscosity, with more plastic clay as the main raw material, is opaque, and has subtle pores and weak absorbance, with the sound of attack turbidity. Porcelain is made of clay, feldspar, and quartz, is translucent, does not absorb water, and has corrosion-resistant, rigid, and tight fetal mass; the sound of its tapping is brittle. Traditional Chinese ceramic arts and crafts have high quality and beauty, with high artistic value, aspects for which it is well known in the world.

The basic raw material of ceramic is composed of rock weathering and decomposition on the surface and belongs to the aqueous aluminum silicate mineral, including quartz, feldspar, porcelain stone, clay, and ceramic raw materials. Among them, clay and kaolin are plastic substances, which have plastic and binding roles in the process of porcelain making, to ensure the strength of the billet body and various practical energy after firing. Quartz is a barren material that reduces viscosity and, therefore, can reduce the viscosity of the billet. The burning part of the quartz is dissolved in the feldspar glass, which can improve the viscosity of the liquid phase, prevent deformation at high temperature, and act as a skeleton frame in the porcelain billet after cooling. Feldspar is a flux raw material. After high-temperature melting, some quartz and ceramic raw materials compounds can be melted and the high viscosity glass can have a high-temperature bonding effect. The main components of the clay are the oxides of silicon and aluminum, coupled with iron, potassium, magnesium, calcium, titanium, and sodium, which together constitute the eight elements of rock making and are also the elements often involved in porcelain making. Among these eight elements, aluminum and silicon are the skeleton materials of ceramics, while potassium, sodium, magnesium, calcium, and alkaline-earth metal oxides belong to melting materials. Iron and titanium oxides have coloring functions in firing white pottery and white porcelain. Before the Song and Yuan Dynasties, porcelain raw materials were made with the one-yuan formula of porcelain stone. After the Song Dynasty, with the improvement of the kiln and the gradual improvement of the firing temperature, the circular point of a single formula became increasingly prominent. The Yuan Dynasty invented the binary formula of porcelain stone + kaolin creatively and improved the aluminum content of the fetus.

Although the porcelain process can be made using a single raw material and transforming it into porcelain, composites of binary ceramic raw materials cannot be used for tire production, because the ceramic raw materials aluminum content is very high, melt content is very low, it is difficult to burn, and difficult to incorporate with porcelain stone, and therefore, improving the micro-structure, the mechanical strength, and thermal stability of the porcelain tire, and reducing defects such as deformation are necessary. As ceramic raw materials has an important role in porcelain making and significant component of Chinese ceramic art, in the creation of contemporary ceramic art, increasing attention should be paid to ceramic raw materials thermal stability to increase the success rate of ceramic art modeling; the fusion of ceramic raw materials and polymer materials is also an area of focus for current researchers and ceramic art creators.

As a natural clay mineral, ceramic raw materials has a layered structure, strong adsorption properties, and good biocompatibility, and has a typical 1:1 layered silicate crystal structure. These characteristics give ceramic raw materials a superior advantage in contemporary ceramic art. This advantage is especially evident in architectural ceramic art in terms of disguised thermal storage performance.

Calcination and acid immersion of silica nanoplates of natural-coal-mined ceramic raw materialsite minerals were successfully synthesized so that Ag nanoparticles with small particles, good dispersion, and size of around 6 nm can be evenly attached to the surface of Sn_2_ + activated SNSs, which greatly improves the thermal conductivity and stability of Ag/Sn_2_ + -SNSs. The structural synergy of Ag/Sn_2_ + -SNSs serves to improve the thermal conductivity of the phase transition materials. The introduction of Agn Ps significantly shortens the melting and solidification cycles of materials and promotes the heat transfer of composite phase transition materials. Moreover, the composite phase transition material can still maintain good thermal reliability after 202 cycles, fully indicating its application potential in heat storage systems [15]. The phase transition properties formed by the fusion of ceramic raw materials with other polymer materials are shown in Table 1.

**Figure 1:**
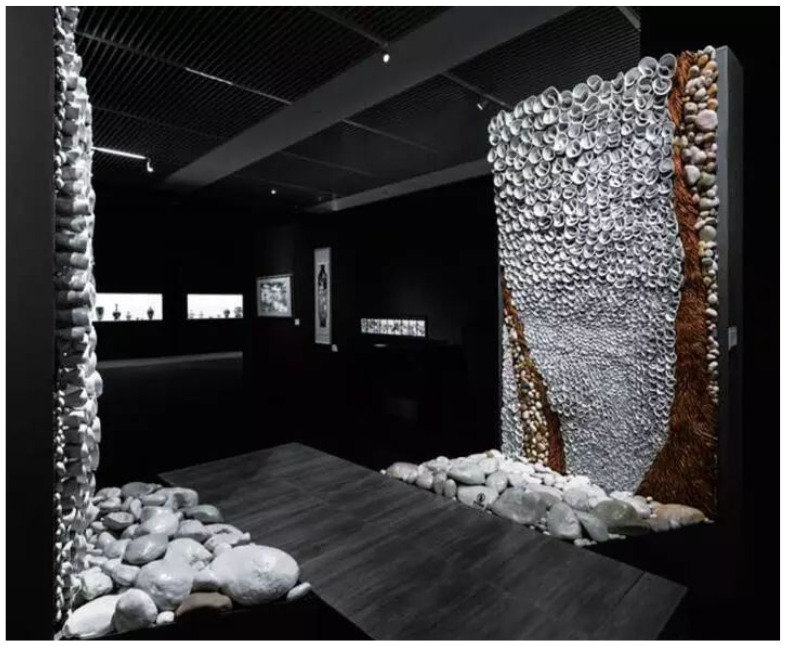
“The Box of Life” by Zhu Legend, a contemporary ceramic artist.

According to the phase transition performance of several materials in the table, the fusion material has good heat storage, ventilation, and air conditioning functions, suitable for its use in ceramic heating, ventilation, and air conditioning, which makes the application of ceramics no longer stay at the level of its use in floor tiles but be extended to applications of a broader space, such as indoor murals, background wall, the wall device of a concert hall, etc. Ceramic raw materials with polymer and polymer composite properties are adopted by ceramists to enhance energy storage; this is most manifest in the contemporary ceramist Zhu Legend ceramic work “The Box of Life” (Figure 1). To improve the toughness of ceramic materials and ceramic artworks and create an interaction between space, sound, light, and color, the potter used a fusion of ceramic raw materials and fatty acids and alcohols on the solid–liquid phase-change material and determined the numerical required according to the ceramic artworks to allocate and maximize the effect of polymer materials in the performance of the ceramic billet. From the perspective of “The Box of Life”, due to the enhancement of toughness of ceramic materials, the creative techniques and properties of ceramics become diverse and more possible. After forming, the works, large and small, with uneven mud pieces rolled into different shapes of flower elements, were installed in the interior space, independent from the building having a mobile art space, changing the use of ceramic as a single building material attribute. The conveyed idea in this artwork is that with the rapid changes of social life, human feelings of the world are changing, but ceramic art began by simple visual appreciation, gradually shifting toward an immersive interactive experience; such an experience is based on the use of mud and the resulting dialogue with nature and humanity, prompting human and space to convey the internal linkage between people and objects. Due to the performance advantage of ceramic raw materials composite with high-polymer material, the strength and toughness of artworks are guaranteed and the thermal stability is improved during firing, thus improving the product yield and the smooth texture, to follow the tradition of Chinese works in integrating the environment, people, light, and sound sources to form a complete space and increasing the tension of the interior space in terms of visual art; it also brings a sense of temperature and ecology to the viewer.

## 4. Incorporating Polymer Materials into the Creation of Modern Ceramics to Enhance the Texture and Artistic Expression of the Porcelain

The ceramic polymer material is a new and rapidly developed thermal protection material [16]. Different from traditional polymer materials, which burn in flame or high-temperature environments, this new material can maintain the polymer’s soft texture, flexibility, and easy-to-form characteristics, and can quickly become ceramic when placed in flame or high-temperature environments; ceramic’s sintering body is hard and has a certain bending strength and compression strength, and as a ceramic material, is flame-and corrosion resistant.

The polymer matrix also influences ceramic polymer composites, with different residual substances, which have a great influence on the formation of ceramic bodies. Alexander et al. prepared ceramic polymer materials with polyethylene, nitrile rubber, and polyvinyl acetate, with aluminum hydroxide, ammonium polyphosphate, and talc as porcelain fillers, characterized the sintering effect with linear shrinkage rate and bending strength [17], and studied the effect of different substrates on the sintering effect of ceramic polymer composites.

**Figure 2:**
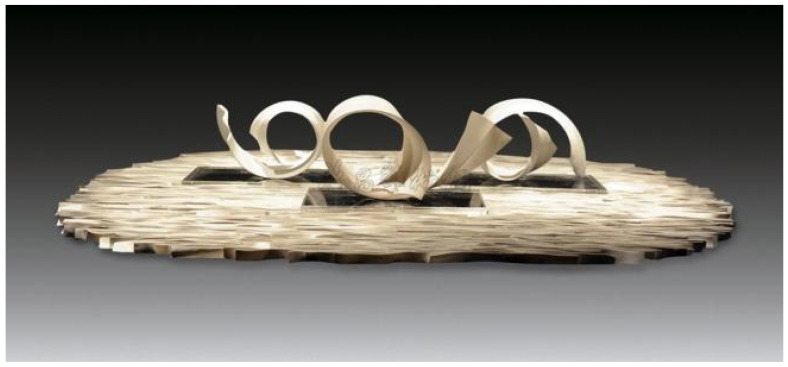
“Solitone” series by Jingjing Zhang.

**Figure 3:**
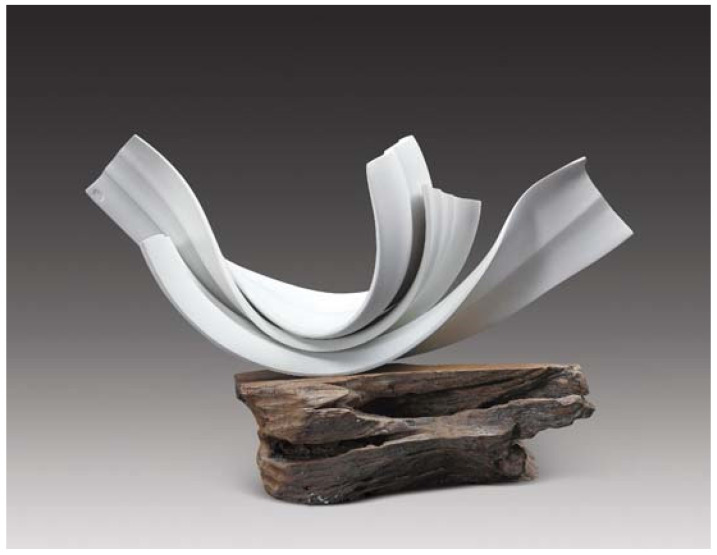
Porcelain Speak New Language series by Jingjing Zhang.

The results showed that the certified polymer material prepared for the substrate had the best sintering performance, with a linear shrinkage rate of −5.6% and bending strength of 5.3 MPa. As an important component of ceramic polymer composition, the number of porcelain additives has a great impact on the ceramic properties formed by ceramic polymer composition ablation. Jianhua, G et al. developed the effect of low melting point in glass powder content used in the porcelain-forming process [18]. The results show that with the increase in glass powder content, the porcelain filling mica and molten glass powder react to generate quartz and magnesium silicate crystals. Within a certain range, with the increasing content of the glass powder, the linear contraction of the ceramic body increases, and the bending strength increases [19]. The co-crystal reaction formed by polymer materials is used to improve the bending strength of ceramic molding, which is the expression technique often used by ceramists in ceramic production. Jingjing Zhang is one of the representative ceramists. In her works, the visual tension of ceramic pottery is often expressed in a curved shape, producing columns of ceramic works, such as those of the “Solitone” series (Figure 2), the “Porcelain Speak New Language” series (Figure 3), etc.

**Figure 4:**
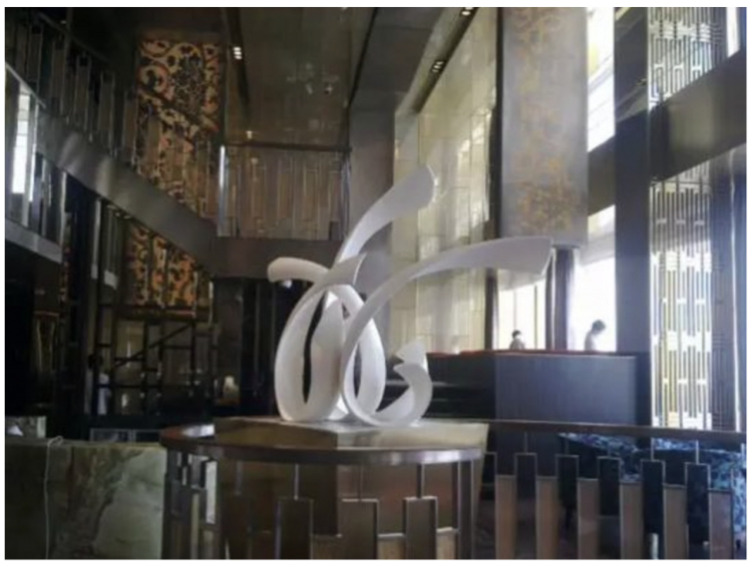
Ceramics installation in hotel furnishing by Jingjing Zhang.

Due to the increased bending strength, ceramics can increase the volume of the work in a public art space to accommodate the visual space of the environment. In addition, many designers, architects, and well-known artists are focusing on ceramics and reenvisioning how ceramics can be applied as a new element. The significance of ceramic intervention in modern space is evident in the space design and art of the Norjin Hotel, which presents a very good model. In the past, among the art forms used in hotels, modern ceramics were ranked after all other art categories. However, the Norjin Hotel introduced massive ceramic works into the environment. At the same time, ceramic elements in other areas are used in different corners of the space. Ceramic artwork intervention in the hotel space setting is not a possibility—it is already underway. However, there are still many technical problems about the degree of intervention. Ceramics is a dominant process industry, and no matter how good the design and modeling, they eventually must pass through fire; in the hotel lobby, massive ceramic elements (Figure 4) are used, after much artistic “suffering”, i.e., they need considerable effort and are labor intensive; this is also owing to the ceramic raw materials added in polymer materials, resulting in a massive volume to be completed.

**Figure 5:**
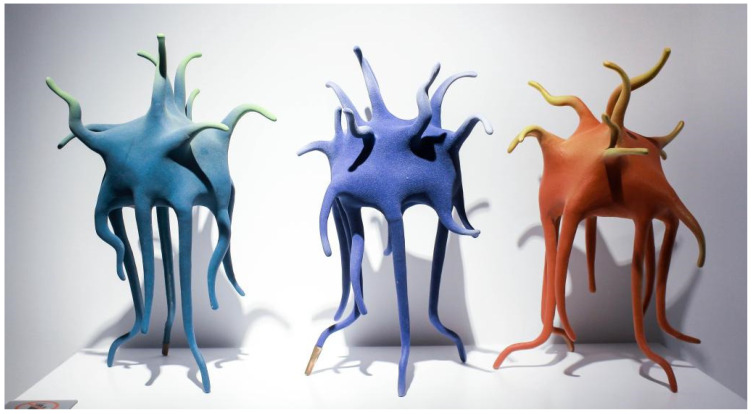
“Language of Life” Qin He, Qingqing Li, and Mengniu Peng.

Haibin et al. [20] studied the effect of the number of ceramic additives on the mechanical strength of ceramic. The results show that the mechanical strength of the ceramic body gradually increases as the content of porcelain aid increases. However, it is not that the higher content of the porcelain aid is more conducive to the formation of porcelain. Under a certain amount of filling, the addition of the porcelain aid helps to improve the percentage of the melting components at high temperatures, thus promoting the porcelain formation reaction between the porcelain filler and the matrix residue. However, if the content of the porcelain aid is too high, there are too many melting components at high temperatures, which makes the composite material lose its supportive performance and lead to severe volume contraction, which is not conducive to the formation of complete ceramics. Therefore, the improvement of ceramic porcelain technology by polymer materials is realized by the use of contemporary ceramic creation. Using this type of porcelain aid to enhance the ultimate strength of the ceramic manifests in the ceramic works of He Qin, Li Qingqing, and Peng Mengniu (Figure 5). For instance, “Language of Life” is very vivid. In the process of presenting the evolution of life, this work shows an abstract, free form of life transformed to the germination stage of the image. As the ceramic material underwent the firing process, water separation was used to cause objects to gradually shrink into several thin support columns, the weight of the main part being obviously supported by them; incorporating porcelain additives into the composite material makes the support pillars strong enough to bear the weight of the main body, effectively solving the challenge of completion of the work for the ceramist while realizing the visual impression of the remarkable evolution of life.

## 5. Gel injection Molding Process Helps 3D Ceramic Printing to Realize the Diversified Vision of Ceramic Works

At present, with the frequent communication between Chinese and Western cultures, contemporary ceramic art creation is not satisfied with a single visual form. Ceramist artists gradually pay attention to the application of mechanical and tonic image structure in ceramic art creation, presenting the virtual and real visual effect of ceramic artworks, to increase the narrative of ceramic artworks. As a better technology, 3D printing technology is widely used in various industries. The ceramic industry also pays attention to the fast, precise, and various forms of 3D printing technology, which can realize mass production in the ceramic industry, with breakthrough artificial mold making and precision accuracy of parameter coefficients. The traditional 3D printing technology is used for ceramic molding, which is mostly used in the manufacturing of solid porcelain tires. This method is difficult to form complex structural porous ceramics. In addition, the long mold production cycle and high costs are also its disadvantages. Therefore, the injection mode of polymer gel and the fusion with a 3D printing mold can solve the molding process of porous ceramic materials. A combination of traditional ceramic molding techniques and 3D molding techniques is used in various steps of the printing process such as making molds in stereo light-curing 3D printing technology, gel injection molding to form complex structural porous ceramics, and finally, removing the resin mold [21,22] to make high-precision regular resin mold using light-curing molding technology; the low viscosity and high solid content alumina ceramic slurry required for the gel injection molding process are optimized, and the aluminum oxide ceramic billet is formed by a vacuum compression process, to achieve the net formation of porous ceramic parts of complex structures.

**Figure 6:**
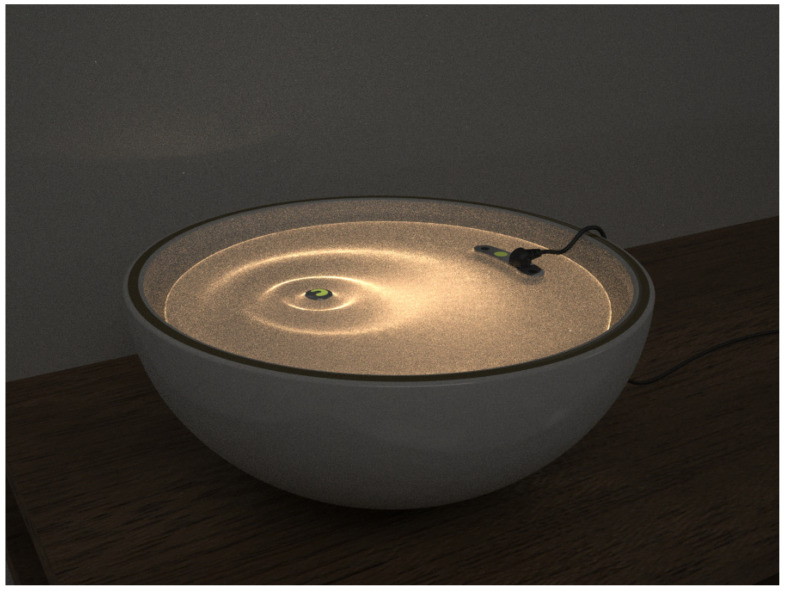
Xiang He by Xiaobing Hu.

For ceramic materials, the processing of its 3D printing technology is more difficult, as there are many unsolved problems—namely, the surface roughness is too large, the mechanical performance is not ideal, the porosity is too large, and the low accuracy of the parts, which have been existing problems. A 3D ceramic printing technology is also difficult to adapt to a variety of materials that often need to develop a corresponding 3D printing technology for a certain characteristic, resulting in high costs. However, with the continuous improvement in both technology and theory, ceramic 3D printing technology has made great progress, which is also a research hot spot and focus of current studies. If the appealing feature of high-temperature sintering used in the traditional ceramic process cannot be achieved by 3D ceramic printing, the modeling technology of 3D ceramic printing can complete complex three-dimensional structures that cannot be realized by the traditional ceramic process. The integration of 3D printing and polymer gel brings opportunities for ceramic creation. People in design and fashion use 3D printing to realize their ideas, also used in ceramic creation, and this trend will continue to grow in the future. The integration of 3D printing and polymer gel facilitates the realization of difficult techniques such as picking, carving, painting, modeling, decorative techniques of traditional ceramic creation, etc. Through the 3D software (3D max 2021(macOS), AUTODESK, Beijing, China) preset, the ceramic form will be well presented to the viewer. This form of creation is more intuitive in the works of ceramic expert Xiaobing Hu, The traditional ceramic lamp theme presented in his work is unfeasible using traditional porcelain-making techniques (Figure 6). According to Anadolu University’s Professor Emre Can, “The nature around us has both material, intuitive feelings and internal structures that affect us. The effect that these structures affect us is the most important factor affecting me. Although nature is so natural, the numbers are very unnatural. My goal is to transform artificial molds from a machine to organic structures with a different touch, capture the opposition between artificial and organic, and deform the ceramic structures produced by the machine to show new forms”.

## 6. Conclusions

To summarize, the involvement of polymer materials in the preparation of ceramic raw materials plays an important role in the creation of daily ceramics and special ceramics but also brings a new experience for modern ceramic art creation. Using polymer materials and ceramic raw materials allows the ceramists to incisively and vividly convey their creative concepts with unconstrained style and, therefore, provides more possibilities for contemporary ceramic art creation. This article mainly discussed the function of ceramic art creation, and the visual image and artistic expression of textures with the use of aspects such as an artistic narrative; the paper also addressed the use of polymer materials in the preparation of mud, for the implementation of the gel injection molding process, effectively solving the problem of realizing fine and multidimensional elements for creating ceramic artworks, as well as the integration of gel injection molding process with 3D printing technology, unfolding new experiences for contemporary ceramic art creation, both in using ceramic artworks in the heart of space in the environment to perfectly render its visual form and to guarantee the airflow and thermal expansion of ceramic artworks in space, effectively showing the superior properties of ceramics. With the progress of technology, the application trend of polymer materials in the field of ceramic art creation will be better demonstrated.

## Figures and Tables

**Table 1:** Thermal properties of fusion of ceramic raw materials and polymer materials.

Composite PCM	Phase Change Temperature/°C	Latent Heat Phase Chang/(J g)
Lauric acid (65%)/expanded perlite	46	92
Lauriccapric acid + fire retardant/gypsum	20	30
Lauricstearic acid (40%)/gypsum	30	50.5
Capricmyristic acid (22%)/VMT	16	27
Capricmyristic acid/expanded perlite	23	88
LA-LAL/ceramic raw materials	22	45

## Data Availability

The data presented in this study are available on request from the corresponding author.
